# Paracoccidioidomycosis due to *Paracoccidioides brasiliensis* S1 plus HIV co-infection

**DOI:** 10.1590/0074-02760170310

**Published:** 2018-03

**Authors:** Priscila Marques de Macedo, Rodrigo Almeida-Paes, Marcos de Abreu Almeida, Rowena Alves Coelho, Hugo Boechat Andrade, Ana Beatriz Teixeira Brandão Camello Ferreira, Rosely Maria Zancopé-Oliveira, Antonio Carlos Francesconi do Valle

**Affiliations:** 1 Fundação Oswaldo Cruz-Fiocruz, Instituto Nacional de Infectologia Evandro Chagas, Laboratório de Pesquisa Clínica em Dermatologia Infecciosa, Rio de Janeiro, RJ, Brasil; 2 Fundação Oswaldo Cruz-Fiocruz, Instituto Nacional de Infectologia Evandro Chagas, Laboratório de Micologia, Rio de Janeiro, RJ, Brasil; 3 Fundação Oswaldo Cruz-Fiocruz, Instituto Nacional de Infectologia Evandro Chagas, Departamento de Assistência a Pacientes Internados, Fiocruz, Rio de Janeiro, RJ, Brasil

**Keywords:** paracoccidioidomycosis, *Paracoccidioides brasiliensis* S1, HIV, AIDS, co-infection

## Abstract

**BACKGROUND:**

Paracoccidioidomycosis (PCM) is one of the most important systemic mycoses in Latin America and the leading fungal cause of mortality in non-immunosuppressed individuals in Brazil. However, HIV/PCM co-infection can increase the clinical severity in these co-infected patients. This co-infection is rarely reported in the literature mainly because of the different epidemiological profiles of these infections. Furthermore, PCM is a neglected and non-notifiable disease, which may underestimate the real importance of this disease. The advent of molecular studies on the species of the genus *Paracoccidioides* has expanded the knowledge regarding the severity and the clinical spectrum in PCM. In this context, the development of studies to describe the association of the *Paracoccidioides* phylogenetic cryptic species in vulnerable populations, such as HIV-infected patients, appears relevant.

**OBJECTIVE:**

To describe the clinical, epidemiological, therapeutic and prognostic aspects in HIV/PCM co-infected patients, along with the molecular identification of the *Paracoccidioides* species involved in these cases.

**METHODS:**

The investigators performed a molecular and clinical retrospective study involving HIV/PCM co-infected patients, from a reference centre for PCM care in the endemic area of Rio de Janeiro, Brazil, from 1998 to 2015. Molecular identification of the fungal strains was done by amplification of partial sequences of *arf* and *gp43* genes.

**FINDINGS:**

Of 89 patients diagnosed with PCM by fungal isolation in the culture, a viable isolate was recovered for molecular analysis from 44 patients. Of these 44 patients, 28 (63.6%) had their serum samples submitted for enzyme immunoassay tests for screening of HIV antibodies, and 5 (17.9%) had a positive result. All cases were considered severe, with a variable clinical presentation, including mixed, acute/subacute clinical forms and a high rate of complications, requiring combination therapy. *Paracoccidioides brasiliensis* S1 was the species identified in all cases.

**CONCLUSIONS:**

HIV/PCM co-infection can change the natural history of this fungal disease. The authors reinforce the need to include HIV screening diagnostic tests routinely for patients with PCM.

According to the World Health Organization (WHO), around 36.7 million people were living with HIV at the end of 2015 (WHO 2015). In Brazil, for the same year, The Joint United Nations Programme on HIV and AIDS (UNAIDS) revealed that around 830,000 people were living with HIV [UNAIDS (Available from: http://unaids.org.br/estatisticas)]. Since the beginning of the AIDS pandemic, about 35 million people have died because of HIV worldwide (WHO 2015). Fungal infections are potentially lethal AIDS-related illnesses, accounting for more than 700,000 deaths annually, while tuberculosis accounts for about 360,000 (Denning 2016). Despite the importance of these data, most fungal diseases are neglected and do not receive adequate financial support to promote advances in prevention, diagnosis, and treatment (Rodrigues 2016).

Paracoccidioidomycosis (PCM) is endemic in Latin America, and it is the leading cause of death among all systemic mycoses in Brazil, where 80% of PCM cases occur (Coutinho et al. 2002). HIV and PCM co-infection (HIV/PCM) is rarely reported in the literature because of its relatively low frequency, probably due to routine pneumocystosis chemoprophylaxis using co-trimoxazole, which is also effective against *Paracoccidioides* spp., or even because of the protective effect of azoles prescribed to treat and prevent candidiasis or other opportunistic mycoses (Marques et al. 2000). Most importantly, HIV and PCM have different epidemiological profiles (Marques et al. 2000). However, an increase in the burden of HIV/PCM is expected as a result of the HIV/AIDS to increase in interior regions occurring since 2005 in Brazil (MS 2015), and the occurrence of PCM in patients born and raised in urban environments (de Macedo et al. 2016b). The overture of new rural frontiers in the southern region of the country and towards the Amazon rainforest can also promote an increment in these co-infection cases. The enzyme immunoassay tests for screening of anti-HIV antibodies is recommended for patients with suggestive epidemiology and with acute/subacute or mixed clinical forms of PCM according to the Brazilian guidelines for the clinical management of PCM (Shikanai-Yasuda et al. 2017).

Despite the clinical relevance of the newly described *Paracoccidioides* phylogenetic species (Teixeira et al. 2014a), there are no clinical descriptions of their behaviour in the HIV population in the literature. The present work aimed to study HIV/PCM cases from a reference centre in the endemic area of PCM in Rio de Janeiro, with an association of clinical, epidemiological, and prognostic data regarding the *Paracoccidioides* phylogenetic species involved in the cases.

## MATERIALS AND METHODS

*Study design and casuistry* - A retrospective study, from 1998 to 2015, was conducted in the Evandro Chagas National Institute of Infectious Diseases (INI)/Fiocruz, a reference centre for PCM and HIV in the state of Rio de Janeiro, Brazil. Inclusion criteria were PCM diagnosis by isolation of *Paracoccidioides* spp. in culture (Zancopé-Oliveira et al. 2014) with availability of a viable isolate that could be used for molecular identification at the commencement of the study and availability of an HIV diagnosis according to the Brazilian guidelines (MS 2015).

*Clinical and laboratorial routine evaluation* - All patients completed a standard clinical and laboratorial evaluation at the time of admission, or periodically, as stated in a consensus recommendation for PCM, or according to individual clinical indications (Shikanai-Yasuda et al. 2017). This evaluation consisted of physical examinations, blood tests [haematology, liver and renal function tests, Ouchterlony double immunodiffusion for PCM (ID), hepatitis virus serology, CD4 cell count, and HIV viral load], parasitological stool analysis, acid-fast bacilli and culture of clinical specimens, chest radiography, and other imaging examinations when indicated [brain computerised tomography (CT), abdominal CT or ultrasonography]. The adrenal function was evaluated using the ACTH (Cortrosyn®) stimulation test. Low adrenal reserve was defined as the normal basal level failing to reach at least 20 mg/dL in two separate measurements after 30 and 60 min of stimulation.

*Molecular identification of fungal isolates* - Molecular identification of the *Paracoccidioides* strains was obtained by partial DNA sequencing of two protein-coding genes (*arf* and *gp43*) as previously described (de Macedo et al. 2016b).

*PCM and HIV treatment* - Treatment was based on consensus recommendations (Shikanai-Yasuda et al. 2017). Sulfamethoxazole/trimethoprim (SMZ/TMP), itraconazole (ITZ), or amphotericin B (AMB) was the standard drug therapy prescribed. Combination therapy and other drugs, such as fluconazole (FCZ) or terbinafine (TBF), were administered in cases of poor clinical response. HIV treatment was according to the Brazilian Guidelines applicable at the time of diagnosis (MS 2015).

*Prognostic analysis for PCM* - The severity grade was based on a standard classification proposed by Mendes et al. (2012). Cure criteria were defined according to the PCM Brazilian guidelines (Shikanai-Yasuda et al. 2017).

*Ethics* - The study was approved by the INI/Fiocruz Research Ethical Committee under the number CAAE 42590515.0.0000.5262.

## RESULTS

**Fig. 1:**
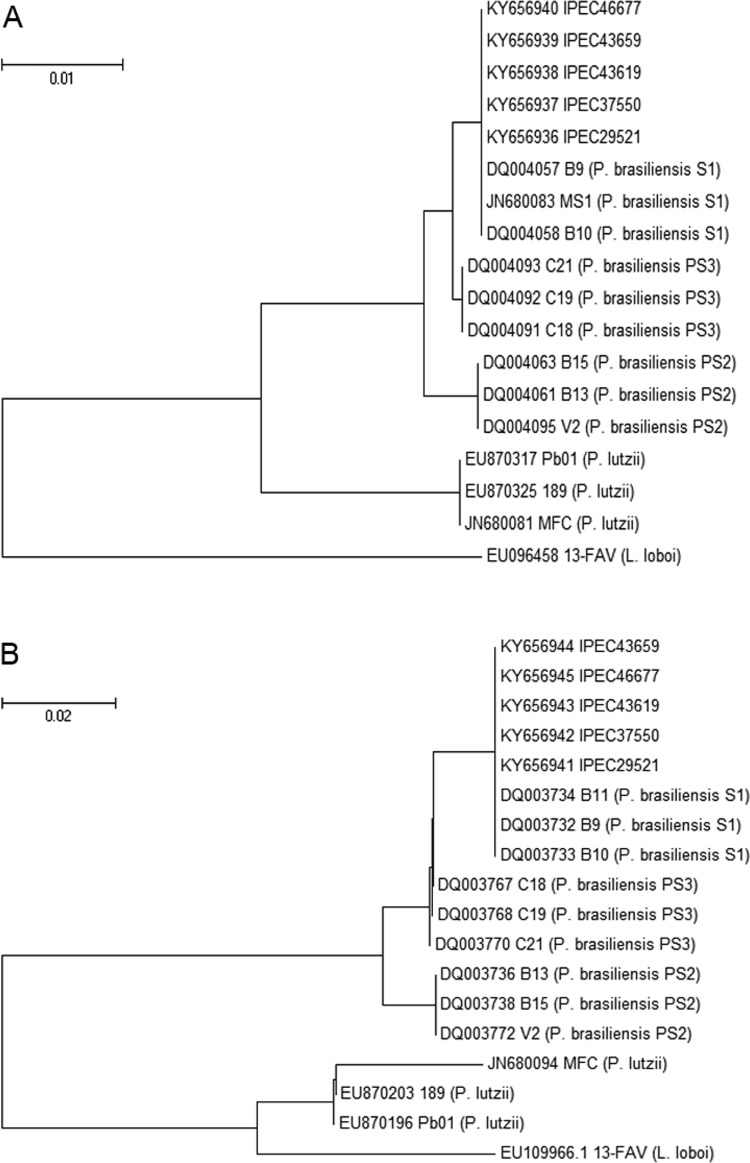
evolutionary relationships of *Paracoccidioides* spp. The evolutionary history was inferred using the Neighbour-Joining method. The optimal trees of *arf* (A) and *gp43* (B) loci are shown. The evolutionary distances were computed using the Maximum Composite Likelihood method and are in the units of the number of base substitutions per site. The evolutionary analyses involved 18 nucleotide sequences and were conducted using the MEGA6 software.

**Fig. 2:**
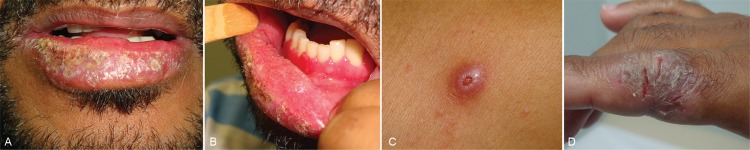
mixed clinical aspects in a HIV/paracoccidioidomycosis co-infected patient from this study. Chronic adult-type manifestations (A-B); acute disseminated skin lesions (C-D).

Of 89 patients diagnosed with PCM by fungal isolation in culture from 1998 to 2015, a viable isolate was recovered for molecular analysis from 44. Of these 44 patients, 28 (63.6%) had their serum samples submitted for enzyme immunoassay tests for screening of HIV antibodies, and five (17.9%) had a positive result. As depicted in Fig. 1, *Paracoccidioides brasiliensis* S1 was the phylogenetic species identified in all cases (GenBankÒ accession numbers KY656936, KY656937, KY656938, KY656939, and KY656940 for *arf* locus; KY656941, KY656942, KY656943, KY656944, and KY656945 for *gp43* locus). The main epidemiological and clinical features characterising these five patients are summarised in Table I. All patients were born in the Brazilian state of Rio de Janeiro, and did not report any previous travel before PCM symptoms began. Occupational profile was not related to rural activities and three patients habited exclusively in urban areas. Mixed forms (Fig. 2) and the acute juvenile PCM form prevailed. The skin and the lymph nodes were the most affected organs. The disease was classified as severe in all cases. The ID was positive in one case. The therapeutic regimen for each patient in this study is detailed in Table II. Combination of drugs were necessary in three patients because of poor clinical responses. The duration of PCM treatment was longer in patients with low CD4 cell count and poor highly active antiretroviral therapy (HAART) adherence. An irregular treatment regimen for PCM was also observed in the same cases of poor HAART adherence. Fatal outcomes occurred in all cases of poor HAART adherence and in a naïve patient in whom PCM behaved as an AIDS-defining illness. Two deaths were related to other HIV-related complications such as neurocryptococcosis and congestive heart failure. One death occurred due to a pseudotumoral brain lesion without microbiological confirmation, treated unsuccessfully as neurotoxoplasmosis. These patients with fatal outcomes did not present with any complications related to PCM. The two surviving patients had serious complications, such as low adrenal reserve and palate perforation, all of them related to PCM. Pulmonary tuberculosis and neurocryptococcosis were observed as co-infections in two patients (Table I).

**TABLE I:** Sociodemographic, epidemiological, clinical, laboratorial, and prognostic data of five patients with HIV/paracoccidioidomycosis (PCM) co-infection from this study

Age	Sex	Occupation	Residence	PCM Clinical form	Disease onset (months)	Organs affected	CD4 / Viral load	Other co-infections	HAART adherence	Fungal isolate from (clinical sample)	PCM serology (immunodiffusion test)	Complications/outcome
25	F	Student	Rural	**Acute** juvenile	5	**Skin, lymph node**	NA	None	Naive	Lymph node aspirate	NP	**Wasting syndrome**/Death (congestive heart failure)
28	M	General services	Urban	Mixed	1	**Skin, lymph node, ADT, lung**	< 200/67,489	Cryptococcosis	Poor	Oral biopsy	Negative	**Wasting syndrome**/Death (neurocryptococcosis)
45	M	Watchman	Urban	Mixed	3	**Skin, lymph node, ADT**	< 200/25,178	Tuberculosis	Poor	Oral biopsy	Positive **(1:128)**	Death (pseudotumoral brain lesion)
52	M	Taxi driver	Urban	Chronic adult	12	ADT	>200/NA	None	Good	Nasal biopsy	Negative	**LAR, PP**/Cured
41	F	Cashier	Rural	**Acute** juvenile	1.5	**Skin, lymph node, liver, spleen**	>200/UD	None	Good	Lymph node aspirate	Negative	**Severe anemia** / Improved

ADT: aerodigestive tract; LAR: low adrenal reserve; PP: palatal perforation; NA: not available; NP: not performed; UD: undetectable. Severity criteria such as multiple organs affected and other aspects are highlighted in bold.

**TABLE II:** Therapeutic characteristics of five patients with HIV/PCM included in this study

	Treatment (months)	
Patient	Initial	Subsequent
1	ITZ 200 mg/day (NA)	None
2	SMZ/TMP 1,600/320 mg/day (56)FCZ 200 mg/day (56)	None
3	SMZ/TMP 1,600/320 mg/day (9)	SMZ/TMP 2,400/480 mg/day (4)*
		SMZ/TMP 1,600/320 mg/day (27)
		SMZ/TMP 2,400/480 mg/day (2)*
		SMZ/TMP 2,400/480 mg/day + TBF 250 mg/day (3)*
		AMB-D 200 mg cumulative dose* - nephrotoxicity
		ITZ 100 mg/day (2)*
		ITZ 200 mg/day (2)*
4	ITZ 200 mg/day (21)	None
5	ITZ 200 mg/day + SMZ/TMP 2,400/480 mg/day (1)	AMB-D 600 mg cumulative dose*
		AMB-L 3,000 mg cumulative dose**
		ITZ 200 mg/day + SMZ/TMP 1,600/320 mg/day (5)
		ITC 100 mg/day (14)

AMB-D: amphotericin B deoxycholate; AMB-L: lypossomal amphotericin B; NA: not available.

* because of low clinical response;

** drug availability.

## DISCUSSION

HIV/PCM co-infection is rarely reported in the literature. The epidemiological profile for this population differs among studies. A case-control study comparing 53 patients with HIV/PCM co-infection, with 106 presenting with classical endemic PCM, found that co-infected patients were less involved in agricultural occupation, whereas our results showed an exclusive urban profile (Morejón et al. 2009). However, another study of 10 HIV/PCM cases showed an epidemiological profile similar to that of the classical endemic PCM (Silva-Vergara et al. 2003).

With regards to clinical aspects, the literature demonstrates a more serious outcome resulting from immunosuppression, with occurrence of disseminated and mixed forms, thereby presenting with clinical signs of both chronic and acute disease, challenging diagnosis, and clinical classification (Almeida et al. 2017, Shikanai Yasuda 2017). Antibodies against *Paracoccidioides* spp. are usually not detected because of the patients' immunosuppression or severe and acute clinical presentations; therefore the diagnosis should not be ruled out for patients in these clinical conditions whose serologic test results are negative (Goldani & Sugar 1995, Bellissimo-Rodrigues et al. 2010, Shikanai-Yasuda et al. 2017). Surprisingly, the unique ID positive result in the cases described herein was from a patient presenting with the mixed clinical form and not the PCM chronic type, as would be expected.

Serious complications such as those described in this study are frequent in patients with HIV/PCM coinfection (Morejón et al. 2009). Adrenal impairment is a common and complication of severe PCM and requires long-term corticosteroid replacement therapy (Tobón et al. 2010). Palatal or nasal perforation is an infrequent but important aesthetic and disabling permanent sequela of chronic-type PCM (Castro et al. 2001). PCM is not usually the main cause of mortality in HIV patients and the lethality in co-infected patients is similar to that in patients without co-infection (Morejón et al. 2009). In this study, most deaths were not related to PCM although in one case it was not possible to rule out PCM as a possible cause of death since neurologic PCM can also be presented as a pseudotumoral brain lesion (Alves et al. 2011).

Regarding therapeutics for PCM, drug combination is an important strategy in patients with HIV/PCM coinfection because of the severity and the refractoriness in these cases, particularly when there is a low treatment adherence. These cases usually require a longer period of treatment, as shown in Table II, for patients 2 and 3. Therefore, patient 3 required frequent changes in his therapeutic regimen because of a low clinical response. This could have been due to an inadequate cell-mediated immune response in this patient, as his CD4 cell count could not be increased, and disease control in PCM depends largely on the cellular immune response (Shikanai-Yasuda et al. 2017). Another hypothesis for the low clinical response in this case was an intrinsic SMZ/TMP parasitic resistance. The attempt to associate TBF to SMZ/TMP in this case was reinforced by previous studies that showed *in vitro* activity of TBF against *Paracoccidioides* (Hahn et al. 2002) and an adequate clinical response to this allylamine in a case of refractoriness to SMZ/TMP in a patient with no immunosuppression (Ollague et al. 2000).

To our knowledge, there are no clinical reports of patients with HIV/PCM coinfection in the literature that include the molecular identification of the fungal strains. Considering the state-of-the-art molecular studies of the etiological agents, their different virulence profiles, the differential host-pathogen interactions, and finally their consequences in clinical presentation, therapeutic response, prognosis as well as diagnostic accuracy, this subject is an increasingly important area for future studies in the PCM field (Hahn et al. 2014, Teixeira et al. 2014a, b, Vidal et al. 2014, de Macedo et al. 2016b). The association of a highly virulent species such as *P. brasiliensis* S1 (Scorzoni et al. 2015) with HIVimmunosuppressed patients would explain the severe disease observed in all patients. For instance, although palate perforation is reported as a rare complication in PCM (Castro et al. 2001), we saw one occurrence of this sequela in the small casuistic herein studied. The extension of this observation to other *Paracoccidioides* phylogenetic species is imperative for better clinical management of this at-risk population.

There is a paucity of studies about phylogenetic species of *Paracoccidioides* in Rio de Janeiro. Until now, there have been four reports on eight clinical cases with phylogenetic analyses of the etiological agent in this region. In one case, *P. brasiliensis* PS2 was reported (de Macedo et al. 2016a) and in the remaining cases *P. brasiliensis* S1 was detected, all in non-HIV infected patients (de Macedo et al. 2016b, 2017a, b). Together with this study, it appears that *P. brasiliensis* S1 is the most prevalent species in Rio de Janeiro, both in non-HIV and HIV groups. However, in order to confirm this hypothesis, more studies are still necessary.

HIV/PCM can change the natural history, the clinical presentation and the therapeutic response of this fungal disease. Whether PCM should be considered as an HIV-predictor disease (Benard & Duarte 2000, Silva-Vergara et al. 2003, Marques 2012), remains to be proven by larger clinical studies. The authors reinforce the existing recommendations to include HIV screening diagnostic tests routinely to all patients with PCM.
